# Supplementary material for: Experiences with implementation of continuous positive airway pressure for neonates and infants in low-resource settings: A scoping review
Source: PLoS One. 2021 Jun 11;16(6):e0252718. doi: 10.1371/journal.pone.0252718 (PMC8195417; doi:10.1371/journal.pone.0252718)
Supplement: S2 File — (DOCX) [file pone.0252718.s002.docx]

# S2. Database Search Queries

MEDLINE 362

Embase 546

Web of Science 260

CINAHL 177

Global Health 330

WHO Global Index Medicus 602

Total 2277

After removing duplicate records 1380

**MEDLINE (Ovid)**

Ovid MEDLINE(R) and Epub Ahead of Print, In-Process & Other Non-Indexed Citations, Daily and Versions(R) 1946 to March 06, 2020

20200309

362 Records

(exp infant/ OR exp "intensive care units, neonatal"/ OR exp neonatology/ OR infant*.ab,kw,ti OR newborn*.ab,kw,ti OR neonat*.ab,kw,ti OR premature.ab,kw,ti OR preterm.ab,kw,ti OR pre term.ab,kw,ti OR baby.ab,kw,ti OR babies.ab,kw,ti OR child*.ab,kw,ti OR bubble.ab,kw,ti)

AND

(exp continuous positive airway pressure/ OR continuous positive airway pressure.ab,kw,ti OR distending airway pressure.ab,kw,ti OR positive pressure ventilation.ab,kw,ti OR positive pressure breathing.ab,kw,ti OR positive expiratory pressure.ab,kw,ti OR positive end expiratory pressure.ab,kw,ti OR cpap.ab,kw,ti OR ncpap.ab,kw,ti OR cppv.ab,kw,ti OR cppb.ab,kw,ti OR bcpp.ab,kw,ti)

AND

(exp developing countries/ OR ((developing OR less developed OR third world OR under developed OR middle income OR low income OR underserved OR under served OR deprived OR poor*) adj1 (count* OR nation* OR state* OR population* OR area* OR economy OR economies)).ab,kw,ti OR (lmic OR lmics).ab,kw,ti OR (resource* adj2 (poor OR limiting OR limited OR low OR constrain*)).ab,kw,ti OR exp africa/ OR exp asia/ OR exp south america/ OR exp latin america/ OR exp central america/ OR (africa OR asia OR south america* OR latin america* OR central america* OR afghanistan* OR albania* OR algeria* OR angola* OR argentina* OR armenia* OR azerbaijan* OR bangladesh* OR belarus* OR belize* OR benin* OR bhutan* OR bolivia* OR bosnia* OR botswana* OR brazil* OR bulgaria* OR burkin* OR burundi* OR cabo verd* OR cape verd* OR cambodia* OR cameroon* OR central african republic OR chad* OR china* OR colombia* OR comoros* OR comorian* OR congo* OR costa rica* OR cote divoire* OR ivorian* OR cuba* OR democratic peoples republic of korea OR djibouti* OR dominica* OR ecuador* OR egypt* OR el salvador* OR salvadoran* OR eritrea* OR eswatini* OR ethiopia* OR fiji* OR gabon* OR gambia* OR gaza OR georgia* OR ghana* OR grenada* OR grenadines* OR guatemala* OR guinea* OR guyana* OR haiti* OR herzegovina* OR hondura* OR india* OR indonesia* OR iran* OR iraq* OR ivory coast* OR jamaica* OR jordan* OR kazakh* OR kenya* OR kiribati* OR kosovo* OR kyrgyz* OR lao OR laoatian* OR lebanon* OR lebanese OR lesotho* OR liberia* OR libya* OR macedonia* OR madagascar* OR malawi* OR malaysia* OR maldiv* OR mali OR malian* OR marshall island* OR mauritania* OR mauriti* OR mexico OR mexican* OR micronesia* OR moldova* OR mongolia* OR montenegr* OR morocc* OR mozambi* OR myanmar* OR namibia* OR nauru* OR nepal* OR nicaragua* OR niger* OR pakistan* OR papua* OR paraguay* OR peru* OR philippines* OR philippino* OR principe OR romania* OR russia* OR rwanda* OR saint lucia* OR saint vincent* OR samoa* OR samoa* OR sao tome* OR senegal* OR serbia* OR sierra leone* OR solomon island* OR somalia* OR south africa* OR south korea* OR sri lanka* OR st lucia* OR st vincent* OR sudan* OR surinam* OR syria* OR tajik* OR tanzania* OR thai* OR timor* OR togo* OR tonga* OR tunisia* OR turkey* OR turk* OR tuvalu* OR uganda* OR ukraine* OR uzbek* OR vanuatu* OR venezuela* OR vietnam* OR west bank* OR yemen* OR zambia* OR zimbabw*).ab,kw,ti)

**Embase (1974-; Elsevier)**

20200309

546 records

('infant'/exp OR 'preschool child'/exp OR 'neonatal intensive care unit'/exp OR infant*:ab,ti OR newborn*:ab,ti OR neonat*:ab,ti OR premature:ab,ti OR preterm:ab,ti OR 'pre term':ab,ti OR baby:ab,ti OR babies:ab,ti OR child*:ab,ti OR bubble:ab,ti)

AND

('positive end expiratory pressure'/exp OR 'CPAP device'/exp OR 'continuous positive airway pressure':ab,ti OR 'distending airway pressure':ab,ti OR 'distending airways pressure':ab,ti OR 'positive pressure ventilation':ab,ti OR 'positive pressure breathing':ab,ti OR 'positive expiratory pressure':ab,ti OR 'positive end expiratory pressure':ab,ti OR cpap:ab,ti OR ncpap:ab,ti OR cppv:ab,ti OR cppb:ab,ti OR bcpp:ab,ti)

AND

('developing country'/exp OR ((developing OR 'less developed' OR 'third world' OR 'under developed' OR 'middle income' OR 'low income' OR underserved OR 'under served' OR deprived OR poor*) NEAR/1 (count* OR nation* OR state* OR population* OR area*)):ab,ti OR lmic:ab,ti OR lmics:ab,ti OR (resource* NEAR/2 (poor OR limiting OR limited OR low OR constrain*)):ab,ti OR africa/exp OR asia/exp OR 'south and central america'/exp OR africa:ab,ti OR asia:ab,ti OR 'south america*':ab,ti OR 'latin america*':ab,ti OR 'central america*':ab,ti OR afghanistan*:ab,ti OR albania*:ab,ti OR algeria*:ab,ti OR angola*:ab,ti OR argentina*:ab,ti OR armenia*:ab,ti OR azerbaijan*:ab,ti OR bangladesh*:ab,ti OR belarus*:ab,ti OR belize*:ab,ti OR benin*:ab,ti OR bhutan*:ab,ti OR bolivia*:ab,ti OR bosnia*:ab,ti OR botswana*:ab,ti OR brazil*:ab,ti OR bulgaria*:ab,ti OR burkin*:ab,ti OR burundi*:ab,ti OR 'cabo verd*':ab,ti OR 'cape verd*':ab,ti OR cambodia*:ab,ti OR cameroon*:ab,ti OR 'central african republic':ab,ti OR chad*:ab,ti OR china*:ab,ti OR colombia*:ab,ti OR comoros*:ab,ti OR comorian*:ab,ti OR congo*:ab,ti OR 'costa rica*':ab,ti OR 'cote d ivoire*':ab,ti OR ivorian*:ab,ti OR cuba*:ab,ti OR 'democratic peoples republic of korea':ab,ti OR djibouti*:ab,ti OR dominica*:ab,ti OR ecuador*:ab,ti OR egypt*:ab,ti OR 'el salvador*':ab,ti OR salvadoran*:ab,ti OR eritrea*:ab,ti OR eswatini*:ab,ti OR ethiopia*:ab,ti OR fiji*:ab,ti OR gabon*:ab,ti OR gambia*:ab,ti OR gaza:ab,ti OR georgia*:ab,ti OR ghana*:ab,ti OR grenada*:ab,ti OR grenadines*:ab,ti OR guatemala*:ab,ti OR guinea*:ab,ti OR guyana*:ab,ti OR haiti*:ab,ti OR herzegovina*:ab,ti OR hondura*:ab,ti OR india*:ab,ti OR indonesia*:ab,ti OR iran*:ab,ti OR iraq*:ab,ti OR 'ivory coast*':ab,ti OR jamaica*:ab,ti OR jordan*:ab,ti OR kazakh*:ab,ti OR kenya*:ab,ti OR kiribati*:ab,ti OR kosovo*:ab,ti OR kyrgyz*:ab,ti OR lao:ab,ti OR laoatian*:ab,ti OR lebanon*:ab,ti OR lebanese:ab,ti OR lesotho*:ab,ti OR liberia*:ab,ti OR libya*:ab,ti OR macedonia*:ab,ti OR madagascar*:ab,ti OR malawi*:ab,ti OR malaysia*:ab,ti OR maldiv*:ab,ti OR mali:ab,ti OR malian*:ab,ti OR 'marshall island*':ab,ti OR mauritania*:ab,ti OR mauriti*:ab,ti OR mexico:ab,ti OR mexican*:ab,ti OR micronesia*:ab,ti OR moldova*:ab,ti OR mongolia*:ab,ti OR montenegr*:ab,ti OR morocc*:ab,ti OR mozambi*:ab,ti OR myanmar*:ab,ti OR namibia*:ab,ti OR nauru*:ab,ti OR nepal*:ab,ti OR nicaragua*:ab,ti OR niger*:ab,ti OR pakistan*:ab,ti OR papua*:ab,ti OR paraguay*:ab,ti OR peru*:ab,ti OR philippines*:ab,ti OR philippino*:ab,ti OR principe:ab,ti OR romania*:ab,ti OR russia*:ab,ti OR rwanda*:ab,ti OR 'saint lucia*':ab,ti OR 'saint vincent*':ab,ti OR samoa*:ab,ti OR samoa*:ab,ti OR 'sao tome*':ab,ti OR senegal*:ab,ti OR serbia*:ab,ti OR 'sierra leone*':ab,ti OR 'solomon island*':ab,ti OR somalia*:ab,ti OR 'south africa*':ab,ti OR 'south korea*':ab,ti OR 'sri lanka*':ab,ti OR 'st lucia*':ab,ti OR 'st vincent*':ab,ti OR sudan*:ab,ti OR surinam*:ab,ti OR syria*:ab,ti OR tajik*:ab,ti OR tanzania*:ab,ti OR thai*:ab,ti OR timor*:ab,ti OR togo*:ab,ti OR tonga*:ab,ti OR tunisia*:ab,ti OR turkey*:ab,ti OR turk*:ab,ti OR tuvalu*:ab,ti OR uganda*:ab,ti OR ukraine*:ab,ti OR uzbek*:ab,ti OR vanuatu*:ab,ti OR venezuela*:ab,ti OR vietnam*:ab,ti OR 'west bank*':ab,ti OR yemen*:ab,ti OR zambia*:ab,ti OR zimbabw*:ab,ti)

NOT

('conference abstract'/it OR 'conference review'/it)

**Web of Science (Clarivate Analytics)**

Indexes=SCI-EXPANDED, SSCI, A&HCI, CPCI-S, CPCI-SSH, BKCI-S, BKCI-SSH, ESCI, CCR-EXPANDED, IC Timespan=All years

20200309

260 Records

TS=("infant*" OR "newborn*" OR "neonat*" OR "premature" OR "preterm" OR "pre term" OR "baby" OR "babies" OR "child*" OR "bubble")

AND

TS=("continuous positive airway pressure" OR "distending airway pressure" OR "distending airways pressure" OR "positive pressure ventilation" OR "positive pressure breathing" OR "positive expiratory pressure" OR "positive end expiratory pressure" OR "cpap" OR "ncpap" OR "cppv" OR "cppb" OR "bcpp")

AND

TS=((("developing" OR "less developed" OR "third world" OR "under developed" OR "middle income" OR "low income" OR "underserved" OR "under served" OR "deprived" OR "poor*") NEAR/1 ("count*" OR "nation*" OR "state*" OR "population*" OR "area*))" OR "lmic" OR "lmics" OR "(resource* NEAR/2 (poor" OR "limiting" OR "limited" OR "low" OR "constrain*")) OR "africa" OR "asia" OR "south america*" OR "latin america*" OR "central america*" OR "afghanistan*" OR "albania*" OR "algeria*" OR "angola*" OR "argentina*" OR "armenia*" OR "azerbaijan*" OR "bangladesh*" OR "belarus*" OR "belize*" OR "benin*" OR "bhutan*" OR "bolivia*" OR "bosnia*" OR "botswana*" OR "brazil*" OR "bulgaria*" OR "burkin*" OR "burundi*" OR "cabo verd*" OR "cape verd*" OR "cambodia*" OR "cameroon*" OR "central african republic" OR "chad*" OR "china*" OR "colombia*" OR "comoros*" OR "comorian*" OR "congo*" OR "costa rica*" OR "cote d ivoire*" OR "ivorian*" OR "cuba*" OR "democratic peoples republic of korea" OR "djibouti*" OR "dominica*" OR "ecuador*" OR "egypt*" OR "el salvador*" OR "salvadoran*" OR "eritrea*" OR "eswatini*" OR "ethiopia*" OR "fiji*" OR "gabon*" OR "gambia*" OR "gaza" OR "georgia*" OR "ghana*" OR "grenada*" OR "grenadines*" OR "guatemala*" OR "guinea*" OR "guyana*" OR "haiti*" OR "herzegovina*" OR "hondura*" OR "india*" OR "indonesia*" OR "iran*" OR "iraq*" OR "ivory coast*" OR "jamaica*" OR "jordan*" OR "kazakh*" OR "kenya*" OR "kiribati*" OR "kosovo*" OR "kyrgyz*" OR "lao" OR "laoatian*" OR "lebanon*" OR "lebanese" OR "lesotho*" OR "liberia*" OR "libya*" OR "macedonia*" OR "madagascar*" OR "malawi*" OR "malaysia*" OR "maldiv*" OR "mali" OR "malian*" OR "marshall island*" OR "mauritania*" OR "mauriti*" OR "mexico" OR "mexican*" OR "micronesia*" OR "moldova*" OR "mongolia*" OR "montenegr*" OR "morocc*" OR "mozambi*" OR "myanmar*" OR "namibia*" OR "nauru*" OR "nepal*" OR "nicaragua*" OR "niger*" OR "pakistan*" OR "papua*" OR "paraguay*" OR "peru*" OR "philippines*" OR "philippino*" OR "principe" OR "romania*" OR "russia*" OR "rwanda*" OR "saint lucia*" OR "saint vincent*" OR "samoa*" OR "samoa*" OR "sao tome*" OR "senegal*" OR "serbia*" OR "sierra leone*" OR "solomon island*" OR "somalia*" OR "south africa*" OR "south korea*" OR "sri lanka*" OR "st lucia*" OR "st vincent*" OR "sudan*" OR "surinam*" OR "syria*" OR "tajik*" OR "tanzania*" OR "thai*" OR "timor*" OR "togo*" OR "tonga*" OR "tunisia*" OR "turkey*" OR "turk*" OR "tuvalu*" OR "uganda*" OR "ukraine*" OR "uzbek*" OR "vanuatu*" OR "venezuela*" OR "vietnam*" OR "west bank*" OR "yemen*" OR "zambia*" OR "zimbabw*")

[excluding] DOCUMENT TYPES: ( MEETING ABSTRACT )

**CINAHL Plus (EBSCO)**

20200309

177 Records

(MH ("Infant+" OR "Intensive Care Units, Neonatal") OR TI (infant* OR newborn* OR neonat* OR premature OR preterm OR pre term OR baby OR babies OR child* OR bubble) OR AB (infant* OR newborn* OR neonat* OR premature OR preterm OR pre term OR baby OR babies OR child* OR bubble))

AND

(MH ("Continuous Positive Airway Pressure") OR TI ("continuous positive airway pressure" OR "distending airway pressure" OR "positive pressure ventilation" OR "positive pressure breathing" OR "positive expiratory pressure" OR "positive end expiratory pressure" OR cpap OR ncpap OR cppv OR cppb OR bcpp) OR AB ("continuous positive airway pressure" OR "distending airway pressure" OR "positive pressure ventilation" OR "positive pressure breathing" OR "positive expiratory pressure" OR "positive end expiratory pressure" OR cpap OR ncpap OR cppv OR cppb OR bcpp))

AND

(MH ("Developing Countries" OR "Africa+" OR "Asia+" OR "South America+" OR "Latin America" OR "Central America+") OR TI (((developing OR "less developed" OR "third world" OR "under developed" OR "middle income" OR "low income" OR underserved OR "under served" OR deprived OR poor*) N1 (count* OR nation* OR state* OR population* OR area* OR economy OR economies)) OR (lmic OR lmics) OR (resource* N2 (poor OR limiting OR limited OR low OR constrain*)) OR africa OR asia OR "south america*" OR "latin america*" OR "central america*" OR afghanistan* OR albania* OR algeria* OR angola* OR argentina* OR armenia* OR azerbaijan* OR bangladesh* OR belarus* OR belize* OR benin* OR bhutan* OR bolivia* OR bosnia* OR botswana* OR brazil* OR bulgaria* OR burkin* OR burundi* OR "cabo verd*" OR "cape verd*" OR cambodia* OR cameroon* OR "central african republic" OR chad* OR china* OR colombia* OR comoros* OR comorian* OR congo* OR "costa rica*" OR "cote divoire*" OR ivorian* OR cuba* OR "democratic peoples republic of korea" OR djibouti* OR dominica* OR ecuador* OR egypt* OR "el salvador*" OR salvadoran* OR eritrea* OR eswatini* OR ethiopia* OR fiji* OR gabon* OR gambia* OR gaza OR georgia* OR ghana* OR grenada* OR grenadines* OR guatemala* OR guinea* OR guyana* OR haiti* OR herzegovina* OR hondura* OR india* OR indonesia* OR iran* OR iraq* OR "ivory coast*" OR jamaica* OR jordan* OR kazakh* OR kenya* OR kiribati* OR kosovo* OR kyrgyz* OR lao OR laoatian* OR lebanon* OR lebanese OR lesotho* OR liberia* OR libya* OR macedonia* OR madagascar* OR malawi* OR malaysia* OR maldiv* OR mali OR malian* OR "marshall island*" OR mauritania* OR mauriti* OR mexico OR mexican* OR micronesia* OR moldova* OR mongolia* OR montenegr* OR morocc* OR mozambi* OR myanmar* OR namibia* OR nauru* OR nepal* OR nicaragua* OR niger* OR pakistan* OR papua* OR paraguay* OR peru* OR philippines* OR philippino* OR principe OR romania* OR russia* OR rwanda* OR "saint lucia*" OR "saint vincent*" OR samoa* OR samoa* OR "sao tome*" OR senegal* OR serbia* OR "sierra leone*" OR "solomon island*" OR somalia* OR "south africa*" OR "south korea*" OR "sri lanka*" OR "st lucia*" OR "st vincent*" OR sudan* OR surinam* OR syria* OR tajik* OR tanzania* OR thai* OR timor* OR togo* OR tonga* OR tunisia* OR turkey* OR turk* OR tuvalu* OR uganda* OR ukraine* OR uzbek* OR vanuatu* OR venezuela* OR vietnam* OR "west bank*" OR yemen* OR zambia* OR zimbabw*) OR AB (((developing OR "less developed" OR "third world" OR "under developed" OR "middle income" OR "low income" OR underserved OR "under served" OR deprived OR poor*) N1 (count* OR nation* OR state* OR population* OR area* OR economy OR economies)) OR (lmic OR lmics) OR (resource* N2 (poor OR limiting OR limited OR low OR constrain*)) OR africa OR asia OR "south america*" OR "latin america*" OR "central america*" OR afghanistan* OR albania* OR algeria* OR angola* OR argentina* OR armenia* OR azerbaijan* OR bangladesh* OR belarus* OR belize* OR benin* OR bhutan* OR bolivia* OR bosnia* OR botswana* OR brazil* OR bulgaria* OR burkin* OR burundi* OR "cabo verd*" OR "cape verd*" OR cambodia* OR cameroon* OR "central african republic" OR chad* OR china* OR colombia* OR comoros* OR comorian* OR congo* OR "costa rica*" OR "cote divoire*" OR ivorian* OR cuba* OR "democratic peoples republic of korea" OR djibouti* OR dominica* OR ecuador* OR egypt* OR "el salvador*" OR salvadoran* OR eritrea* OR eswatini* OR ethiopia* OR fiji* OR gabon* OR gambia* OR gaza OR georgia* OR ghana* OR grenada* OR grenadines* OR guatemala* OR guinea* OR guyana* OR haiti* OR herzegovina* OR hondura* OR india* OR indonesia* OR iran* OR iraq* OR "ivory coast*" OR jamaica* OR jordan* OR kazakh* OR kenya* OR kiribati* OR kosovo* OR kyrgyz* OR lao OR laoatian* OR lebanon* OR lebanese OR lesotho* OR liberia* OR libya* OR macedonia* OR madagascar* OR malawi* OR malaysia* OR maldiv* OR mali OR malian* OR "marshall island*" OR mauritania* OR mauriti* OR mexico OR mexican* OR micronesia* OR moldova* OR mongolia* OR montenegr* OR morocc* OR mozambi* OR myanmar* OR namibia* OR nauru* OR nepal* OR nicaragua* OR niger* OR pakistan* OR papua* OR paraguay* OR peru* OR philippines* OR philippino* OR principe OR romania* OR russia* OR rwanda* OR "saint lucia*" OR "saint vincent*" OR samoa* OR samoa* OR "sao tome*" OR senegal* OR serbia* OR "sierra leone*" OR "solomon island*" OR somalia* OR "south africa*" OR "south korea*" OR "sri lanka*" OR "st lucia*" OR "st vincent*" OR sudan* OR surinam* OR syria* OR tajik* OR tanzania* OR thai* OR timor* OR togo* OR tonga* OR tunisia* OR turkey* OR turk* OR tuvalu* OR uganda* OR ukraine* OR uzbek* OR vanuatu* OR venezuela* OR vietnam* OR "west bank*" OR yemen* OR zambia* OR zimbabw*)

**Global Health (EBSCO)**

20200309

330 Records

(TI (infant* OR newborn* OR neonat* OR premature OR preterm OR pre term OR baby OR babies OR child* OR bubble) OR AB (infant* OR newborn* OR neonat* OR premature OR preterm OR pre term OR baby OR babies OR child* OR bubble))

AND

(TI ("continuous positive airway pressure" OR "distending airway pressure" OR "positive pressure ventilation" OR "positive pressure breathing" OR "positive expiratory pressure" OR "positive end expiratory pressure" OR cpap OR ncpap OR cppv OR cppb OR bcpp) OR AB ("continuous positive airway pressure" OR "distending airway pressure" OR "positive pressure ventilation" OR "positive pressure breathing" OR "positive expiratory pressure" OR "positive end expiratory pressure" OR cpap OR ncpap OR cppv OR cppb OR bcpp))

**WHO Global Index Medicus**

20200309

602 Records

(infant* OR newborn* OR neonat* OR "reciÈn nacido" OR "reciÈn nacida" OR "recÈm-nascido" OR "recÈm-nascida" OR premature OR preterm OR "pre term" OR baby OR babies OR bebe OR bebes OR bubble OR "sello de agua" OR "selo d'·gua")

AND

("continuous positive airway pressure" OR "presiÛn positiva continua " OR "press„o positiva contÌnua" OR "positive pressure ventilation" OR cpap OR ncpap OR cppv OR cppb OR bcpp)
